# Vinpocetine Attenuates Neointimal Hyperplasia in Diabetic Rat Carotid Arteries after Balloon Injury

**DOI:** 10.1371/journal.pone.0096894

**Published:** 2014-05-12

**Authors:** Ke Wang, Li Wen, Wenhui Peng, Hailing Li, Jianhui Zhuang, Yuyan Lu, Baoxin Liu, Xiankai Li, Weiming Li, Yawei Xu

**Affiliations:** 1 Department of Cardiology, Shanghai Tenth People's Hospital, Tongji University School of Medicine, Shanghai, China; 2 Department of Cardiopulmonary Circulation, Shanghai Pulmonary Hospital, Tongji University School of Medicine, Shanghai, China; Baker IDI Heart and Diabetes Institute, Australia

## Abstract

**Background:**

Diabetes exacerbates abnormal vascular smooth muscle cell (VSMC) accumulation in response to arterial wall injury. Vinpocetine has been shown to improve vascular remolding; however, little is known about the direct effects of vinpocetine on vascular complications mediated by diabetes. The objective of this study was to determine the effects of vinpocetine on hyperglycemia-facilitated neointimal hyperplasia and explore its possible mechanism.

**Materials and Methods:**

Nondiabetic and diabetic rats were subjected to balloon injury of the carotid artery followed by 3-week treatment with either vinpocetine (10 mg/kg/day) or saline. Morphological analysis and proliferating cell nuclear antigen (PCNA) immunostaining were performed on day 21. Rat VSMCs proliferation was determined with 5-ethynyl-20-deoxyuridine cell proliferation assays. Chemokinesis was monitored with scratch assays, and production of reactive oxygen species (ROS) was assessed using a 2′,7′-dichlorodihydrofluorescein diacetate (H2DCFDA) flow cytometric assay. Apoptosis was detected by annexin V-FITC/PI flow cytometric assay. Cell signaling was assessed by immunblotting.

**Results:**

Vinpocetine prevented intimal hyperplasia in carotid arteries in both normal (I/M ratio: 93.83 ± 26.45% versus 143.2 ± 38.18%, P<0.05) and diabetic animals (I/M ratio: 120.5 ± 42.55% versus 233.46 ± 33.98%, P<0.05) when compared to saline. The in vitro study demonstrated that vinpocetine significantly inhibited VSMCs proliferation and chemokinesis as well as ROS generation and apoptotic resistance, which was induced by high glucose (HG) treatment. Vinpocetine significantly abolished HG-induced phosphorylation of Akt and JNK1/2 without affecting their total levels. For downstream targets, HG-induced phosphorylation of IκBα was significantly inhibited by vinpocetine. Vinpocetine also attenuated HG-enhanced expression of PCNA, cyclin D1 and Bcl-2.

**Conclusions:**

Vinpocetine attenuated neointimal formation in diabetic rats and inhibited HG-induced VSMCs proliferation, chemokinesis and apoptotic resistance by preventing ROS activation and affecting MAPK, PI3K/Akt, and NF-κB signaling.

## Introduction

Diabetes mellitus increases the risk of atherosclerosis and the incidence of complications from atherosclerosis such as coronary artery disease, stroke and so on. Cardiovascular disease increased the rate of all-cause death nearly three-fold and the rate of cardiovascular death nearly five-fold in subjects with diabetes [1]. Although revascularization through balloon dilatation or stent placemet would ameliorate coronary artery disease, patients with diabetes mellitus experienced worse outcomes than non-diabetic patients [2]. Higher rates of restenosis and repeat revascularization are seen in diabetic patients compared to patients without diabetes [3].

Abnormal neointimal hyperplasia is considered the predominant mechanism in the pathogenesis of postangioplasty restenosis [4]. Patients with diabetes exhibit increased intimal hyperplasia after percutaneous coronary intervention (PCI), which correlates with the degree of hyperglycemia [2]. However, optimal therapies against neointimal hyperplasia in diabetics are limited. Even with the application of drug eluting stents (DES), the adjusted risk of restenosis was higher in patients with DM than in patients without DM (RR: 1.23, 95% confidence interval [CI]: 1.10 to 1.37) [3]. Therefore, developing an agent without significant adverse effects is urgently needed.

Vinpocetine is a chemical derivative of vincamine, an alkaloid extracted from the periwinkle plant, Vinca minor [5]. Since marketed in 1978, vinpocetine has been commonly used in many countries to prevent cognitive impairment and cerebrovascular disorders [6]. To date, no significant side effects, toxicities, or contraindications have been reported at therapeutic doses.

Besides affecting voltage-dependent Na^+^ and Ca^2+^ channels, vinpocetine has been proven to act as a non-selective inhibitor of Ca^2+^/calmodulin-stimulated cyclical nucleotide phosphodiesterase-1 (PDE-1), which largely participates in pathological vascular remodeling [7], [8]. Recent studies have revealed that vinpocetine inhibits tumor necrosis factor-α (TNF-α)-induced NF-κB activation in multiple cell types including VSMCs [9]. It also attenuates Akt/STAT3 signaling and induces apoptosis in breast cancer cells [10]. Furthermore, by suppressing ROS production and ERK1/2 activation induced by Platelet-derived growth factor-BB (PDGF-BB), vinpocetine inhibits proliferation and migration of VSMCs [11], which indicates beneficial effects of vinpocetine on vascular complications in diabetics.

Vinpocetine affects PDE1 activity, attenuates inflammation and induces apoptosis, which may imply that it has the potential to relieve hyperglycemia-magnified vascular remodeling. However, little is known about the direct effect of vinpocetine on postangioplasty restenosis in the setting of diabetes. In this study, we investigated the “in vivo” effect of vinpocetine on neointimal hyperplasia, using the endothelial rubbing model of carotid artery in diabetic rats. In addition, we determined the efficacy and mechanisms of vinpocetine on hyperglycemia-induced VSMCs proliferation, chemokinesis and apoptotic resistance.

## Materials and Methods

### Ethics statement

The in vivo experiments were handled in accordance with the Guide for the Care and Use of Laboratory Animals published by the National Institutes of Health (NIH Publication, 8^th^ Edition, 2011). All the animal related procedures were approved by the Animal Care and Use Committee of Shanghai Tenth People's Hospital.

### Reagents and antibodies

Vinpocetine, streptozotocin powder, D-(+)-Glucose and D-Mannitol were purchased from Sigma-Aldrich (St. Louis, MO, USA). Antibodies to cycling D1, ERK1/2, phospho-ERK1/2, p38, phospho-p38, JNK, phospho-JNK, AKT, phospho-AKT, phospho-IκBα and β-actin were all purchased from Cell Signaling Technology (Beverly, MA, USA). Antibodies to PCNA and Bcl-2 were obtained from Santa Cruz Biotechnology (Santa Cruz, CA, USA). 2′,7′-dichlorodihydrofluorescein diacetate (H2DCFDA) and Click-iT EdU Imaging Kits were purchased from Invitrogen (Carlsbad, CA, USA). Annexin V-FITC/PI flow cytometric assay kit was purchased from BD Biosciences (San Jose, CA, USA). The In Situ Cell Death Detection Kit, POD was purchased from Hoffmann-La Roche (Basel, Switzerland). Rat Insulin ELISA Kit was purchased from Shibayagi (Shibukawa, Gunma, Japan).

### Animal models and experimental design

Diabetic and balloon injury rat models were used to explore the effects of vinpocetine on hyperglycemia-facilitated neointimal hyperplasia in this study. Male Sprague-Dawley (SD) rats weighting 160–180 g were procured from the Shanghai Slac Laboratory Animal Co., Ltd. and housed in standard plastic cages with well-ventilated stainless steel grid tops at room temperature with a 12-hour light/dark cycle. Rats were randomly divided into diabetic or control groups after acclimatization for 1 week.

To establish a diabetic model, rats weighing approximately 200 g were fed a high-fat diet (HFD) (Slacom, Shanghai, China) consisting of 45% fat (percentage of total kcal), 19% protein and 36% carbohydrate ad libitum for the initial 2 weeks. The composition of HFD is presented in Table 1. A low dose of streptozotocin (STZ) (35 mg/kg body weight, dissolved in citrate buffer) was injected intraperitoneally (i.p.) after the 2 weeks of dietary manipulation. Seven days after the STZ administration, rats with non-fasting blood glucose ≥16 mM on two consecutive tests were considered diabetic and selected for balloon injury and further studies. Non-fasting blood glucose was measured on diabetic rats every week to monitor the robustness of this model. The rats with consecutive high blood glucose (≥16 mM) were used in this study. Rats in the control group were fed a standard diet (12% of calories as fat). Dietary regimens were maintained until the end of the study.

**Table 1:** Composition of High-Fat Diet.

Ingredients	Diet (g/kg)
Normal pellet diet	495
Lard	204
Sucrose	150
Casein	123
Premix (Vitamin, mineral et al)	20
Maltodextrin	8

The carotid artery balloon injury was performed in both groups when the diabetic model was induced (i.e., after 3 weeks of dietary manipulation in rats). Rats weighing 280–350 g were anesthetized with sodium pentobarbital (50 mg/kg, i.p.). The left common carotid artery (CCA) and left external carotid artery (ECA) were exposed via a midline cervical incision. A 1.2 mm*6 mm Mini Trek PTCA balloon catheter (Abbott Vascular, Illinois, USA) guided by 0.014 inch Runthrough guiding wire (Abbott Vascular) was inserted into the left ECA through an arteriotomy and advanced to the aortic arch. To perform rubbing of the endothelia of the CCA, the balloon was dilated at 3–5 atm to generate adequate resistance, then it was withdrawn to the ECA before deflation. This procedure was repeated three times, rotating the catheter 90° each time. Heparin (250 IU/kg) was injected through the ECA to prevent thrombosis before and after catheterization. The ECA was permanently ligated after the balloon catheter had been removed and the skin wound was repaired. Sham-operated rats from both groups underwent the same operation except for the catheterization.

Following the operation, rats were randomized immediately to receive intraperitoneal injections of vinpocetine (10 mg/kg,) or saline everyday for the next 3 weeks.

### Collection of blood and analytical methods

After drug administration, blood (4–5 ml) and plasma samples (500 µl) from diabetic or non-diabetic rats that had fasted for 12 h before euthanasia, were collected from the left cardiac ventricle. Fasting glucose, total cholesterol and triglyceride levels were determined by colorimetric enzymatic assay systems (Roche MODULAR P-800, Swiss Confederation). Glycated hemoglobin was measured using an ADAMS-HA8106 analyzer (ARKRAY, Kyoto, Japan). Serum insulin level was measured with an ELISA kit (Shibayagi, Gunma, Japan).

### Histological and morphometric analyses

At the end of experiment, the rats were euthanized by intraperitoneal pentobarbital overdose. The proximal segments of the left common carotid arteries were removed, and fixed overnight in 4% paraformaldehyde (PFA). The vessel segments were then embedded in paraffin and cut into 4 µm sections, which were spaced at 1 mm intervals. At least 5 random sections from each vessel were stained with hematoxylin and eosin (H&E) and analyzed to determine the intimal area (IA) and medial area (MA) and to calculate the intima to media ratio (I/M ratio). Data are presented as means ± S.D. from six animals in each group.

### Immunohistochemistry and TUNEL analysis

Immunohistochemical analysis of PCNA was performed to detect the proliferating cells in the neointima. Briefly, after consecutive steps including deparaffinization, nuclear membrane perforation by triton, antigen retrieval with citrate buffer, endogenous peroxidase deactivation with 3% H_2_O_2_, and antigen block with 5% BSA-PBS, sections were incubated overnight at 4°C with a mouse monoclonal anti-rat PCNA antibody (clone PC10, dilution 1∶200), followed by incubation with biotinylated horseradish peroxidase-conjugated secondary antibody (Gene Tech, Shanghai, China) for 60 minutes at room temperature. Subsequently, the sections were visualized with 3′3-diaminobenzidine solution (DAB kit) (Gene Tech) under an optical microscope.

Apoptotic cell death in the neointima was assayed by the terminal deoxynucleotidyl transferase-mediated dUTP nick end labeling (TUNEL) assay with the “In Situ Cell Death Detection Kit, POD” according to manufacturer's instructions. In brief, sections were deparaffinized, rehydrated and digested by proteinase K method. Then, sections were incubated with fresh 3% hydrogen peroxide for 20 min, washed with PBS, incubated with TUNEL reaction mixture for 1 h at 37°C and finally incubated with anti-fluorescein antibody conjugated with horse-radish peroxidase (POD) and DAB kit solution.

At least five random sections from each animal were stained for mean values, which were expressed as a percentage of positive cells in neointimal area. Data are presented as means ± S.D. from six animals in each group.

### Cell culture

Primary VSMCs were cultured as previously reported [13]. Briefly, VSMCs were isolated from the thoracic aorta of 10-12-week-old male Sprague-Dawley rats using the explants technique. Cells were cultured in Dulbecco's modified Eagle's medium (DMEM, Gibco, NY, USA, contained 5 mM glucose) with 10% fetal bovine serum (FBS) (Gibco, NY, USA) and 1% penicillin/streptomycin at 37°C in 5% CO_2_. Immunocytochemical localization of α-smooth muscle actin and morphology was used to confirm the purity of VSMCs. Cells from passages 3 to 5 were used throughout this study.

For cell experiments, VSMCs were synchronized by serum deprivation for 48 h and then pre-incubated with or without vinpocetine for 1 hour before stimulation with high glucose (10 or 25 mM) in medium containing 0.5% FBS for the indicated time. Normal glucose (5 mM) was used as the control. D-Mannitol was used to balance osmolarity in each well. For all data shown, individual experiments were repeated at least three times.

### Cell proliferation assay

The 5-ethynyl-2′- deoxyuridine (EdU) cell proliferation assay was used to determine the VSMC proliferation. Briefly, VSMCs were incubated with EdU labeling mixture (10 µM) for 24 h during synchronization. The EdU-positive cells were detected strictly according to the manufacturer's instructions (Click-iT EdU Imaging Kits, Invitrogen). Then, the nuclei were stained with Hoechst 33342 (5 µg/ml diluted in PBS). For each well, 5 visual fields were randomly chosen to obtain fluorescence images with a DMI6000 fluorescent microscope (Leica, Germany). Data were expressed as percentage of EdU positive cells compared with total cell numbers.

### Wound healing assay

VSMCs were seeded in 6-well plates with DMEM (normal glucose) supplemented with 10% FBS overnight. After synchronization, confluent cells were scratched with 10 µl pipette tips followed by vinpocetine pre-incubation and subsequent high-glucose stimulation. Photographs were taken at 0, 12, 24 and 48 h after wounding. Quantitative data of the scratch wound assay were analyzed by the gap areas relative to those measured at 0 h after wounding.

### Determination of intracellular reactive oxygen species (ROS)

Intracellular ROS generation was detected by H2DCFDA fluorescent assay using flow cytometry (FACSCalibur, CA, USA). After pretreatment with vinpocetine, VSMCs were incubated with HG and H2DCFDA (10 µM) for 30 minutes. Then, VSMCs were trypsinized, washed, and resuspended in PBS before fluorescence measurement with flow cytometry at excitation of 488 nm and emission of 528 nm. Data were expressed as the percentage of fluorescent positive cells to the total number of cells.

### Apoptosis assay

After synchronization, VSMCs underwent vinpocetine treatment and HG stimulation for another 48 h. The annexin V-FITC/PI flow cytometric assay was performed to determine the proportion of apoptotic cells in different groups. Briefly, harvested cells were resuspended in binding buffer at a concentration of 10^6^ cells per ml, followed by incubation with annexin V-FITC for 20 min and subsequently with PI for 5 min in darkness. At least 10000 stained cells of each sample were analyzed by flow cytometer. Data were analyzed with CELL Quest software (BD Biosciences).

### Western blot assay

Cells were lysed in RIPA buffer with a protease inhibitor mixture (Santa Cruz), and protein concentrations were measured using the BCA protein assay kit (Beyotime, Shanghai, China). Equal amounts of protein were loaded, separated by SDS-PAGE and transferred to nitrocellulose membranes. After blocking with 5% skimmed milk in Tris-buffered saline with tween20 (TBST) at room temperature, the membranes were incubated overnight at 4°C with primary antibodies. After being incubated with the respective secondary antibodies, immune complexes were detected using the Odyssey system (LiCor, Lincoln, NE, USA) based on the protocol. Antibodies were diluted in 5% BSA-TBST at the following concentrations: Cyclin D1 (1∶1000), PCNA (1∶800), ERK1/2 (1∶1000), phospho-ERK1/2 (1∶800), p38 (1∶1000), phospho-p38 (1∶800), JNK (1∶1000), phosphor-JNK (1∶500), AKT (1∶1000), phosphor-AKT (1∶500), phospho- IκBα (1∶800), Bcl-2 (1∶1000) and β-actin (1∶2000).

### Statistical analysis

Continuous variables were presented as mean ± S.D. The statistical significance of the differences was determined by the analysis of variance (ANOVA) or unpaired two-tailed t-tests in SPSS system; a value of P<0.05 was considered significant.

## Results

### Vinpocetine does not affect body weight and metabolism in vivo

The final body weights of rats were lower in the diabetic group than the control group, but they were not influenced by the 3-week treatment with vinpocetine (10 mg/kg/day IP). Circulating levels of glucose, glycated hemoglobin and triglyceride were remarkably increased in both groups of diabetic rats compared to those in the control group; however there was no difference between the saline- and vinpocetine-treated groups (Table 2). In contrast, insulin levels fluctuated in normal range and no discrepancy existed among groups.

**Table 2:** Effects of vinpocetine on body weight, fasting plasma glucose, fasting plasma insulin, glycated hemoglobin, and lipid profiles in non diabetic or diabetic rats.

	Control + saline (n = 12) †	Control + Vinp (n = 12) †	DM + saline (n = 12) †	DM + Vinp (n = 12) †
SBW (g)	203.5 ± 9.32	205.2 ± 10.53	207.1 ± 10.16	204.7 ± 9.91
EBW (g)	432.8 ± 10.15	433.2 ± 11.04	419.4 ± 10.28*	418.8 ±10.03#
FPG (mmol/L)	5.4 ± 0.43	5.6 ±0.39	16.2±2.29*	17.1 ± 2.91#
FPI (ng/mL)	1.79 ± 0.16	1.76 ± 0.15	1.59 ± 0.18	1.61 ± 0.19
GHB (%)	3.83 ± 0.39	3.63 ± 0.42	7.76 ± 0.81*	8.05 ± 1.03#
PTC (mmol/L)	1.44 ± 0.12	1.52 ± 0.13	1.42 ± 0.17	1.49 ± 0.16
PTG (mmol/L)	0.68 ± 0.12	0.65 ± 0.14	1.61 ± 0.15*	1.64 ± 0.17#

Data are presented as the mean ± SD.

The abbreviations denote Vinp, vinpocetine; SBW, start body weight; EBW, end body weight; FPG, fasting plasma glucose; FPI, fasting plasma insulin; GHB, glycated hemoglobin; PTC, plasma total cholesterol; and PTG, plasma triglyceride.

† Six rats underwent balloon injury and the others underwent sham operation.

^*^means p<0.05 vs control + saline group

# means p<0.05 vs control + vinp group.

### Vinpocetine inhibits neointimal formation after balloon injury in vivo

**Figure 1:**
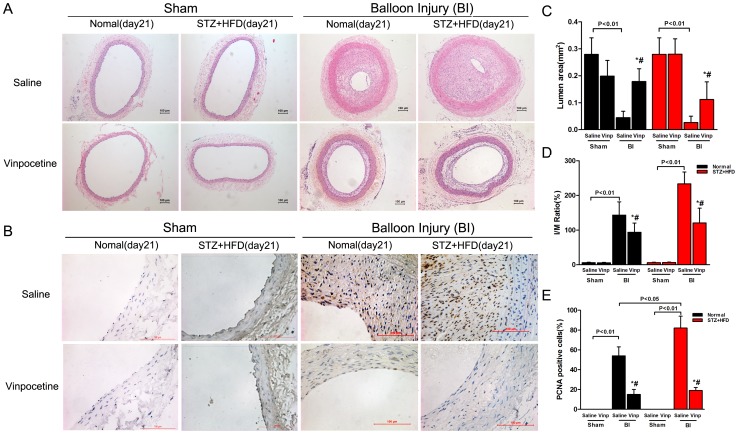
Vinpocetine inhibits neointimal formation after balloon injury in vivo. A–B: Representative microphotographs of hematoxylin and eosin staining (A) and PCNA (brown) immunostaining (B) in sham-operated (Sham) and balloon-injured carotid arteries of diabetic and non-diabetic rats after a 21-day treatment with saline or vinpocetine. Bar  =  100 µm. C–E: Analysis of the lumen area, I/M ratio and PCNA-positive nuclei ratio in the arteries. Data are obtained from six mice from each group and bars indicate means ± SD. Similar results were obtained in three independent experiments. * means P<0.05 compared to Sham; # means P<0.05 compared to Saline.

Nondiabetic and diabetic rats were subjected to balloon angioplasty followed by 3-week of treatment with saline or vinpocetine injections. No rat died during the procedures or during the 21-day treatment with vinpocetine. Representative cross-sections of the carotid arteries from different groups are shown in Figure 1. The neointima was hardly seen in sham operated rats from the diabetic or control groups. Stenosis of the arteries was observed in rats that had undergone balloon injury and was largely aggravated by diabetes, whereas vinpocetine retarded this process of stenosis. Morphologic analysis showed that the I/M ratio was dramatically reduced by vinpocetine in both normal and diabetic animals (93.83 ± 26.45% versus 143.2 ± 38.18% in normal groups, n  =  6, P<0.05; 120.5 ± 42.55% versus 233.46 ± 33.98% in STZ+HFD groups, n  =  6, P<0.05; respectively), as compared with saline-treated groups.

To assess VSMC growth, arterial sections were stained with anti-PCNA antibody and analyzed. Data were expressed as the percentage of PCNA-positive nuclei per neointima. As indicated in Figure 1E, diabetic rats showed a remarkable increase in the percentage of PCNA-positive cells relative to normal rats (P<0.05). The decrease of VSMC proliferation was confirmed by the decrease of PCNA immunostaining intensity in the neointimal layer of carotid arteries from vinpocetine-treated animals, as compared with saline-treated animals.

### Vinpocetine attenuates HG-stimulated VSMCs proliferation and migration

**Figure 2:**
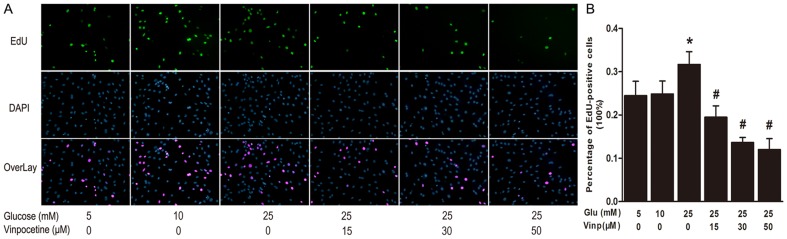
Vinpocetine inhibits VSMCs proliferation stimulated by high glucose. A: VSMCs proliferation was stimulated by normal (5 mM) or higher glucose (10 or 25 mM) in the presence or absence of vinpocetine (15, 30 and 50 µM) for 24 hours. Proliferating VSMCs were detected by EdU incorporation. Nuclei stained by Hoechst 33342 stood for total cell numbers. Data were expressed as percentage of EdU-positive cells compared with total cell numbers. B: Analysis of VSMCs proliferation rates. Data were expressed as percentage of EdU-positive cells compared with total cell numbers. Experiments were performed in triplicate. Bars indicate means ± SD. * means P<0.05 compared to control; # means P<0.05 compared to HG stimulation.

The EdU incorporation assay revealed the effects of vinpocetine on HG-stimulated VSMCs proliferation (Figure 2A). When compared with control samples, the proliferation rate of VSMCs was increased almost 1.3 folds through stimulation with 25 mM glucose for 24 hours (P<0.05). However, DNA replication was significantly blocked by vinpocetine treatment at both lower (15 µΜ) and higher concentrations (30 µM and 50 µM), compared with HG stimulation alone (P<0.05) (Figure 2B).

**Figure 3:**
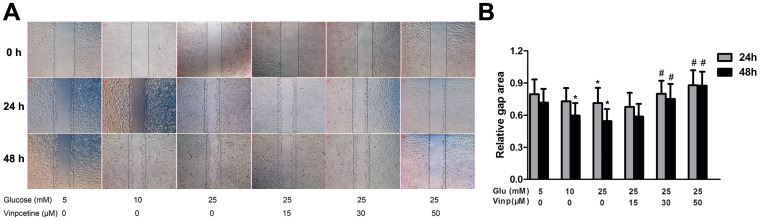
Vinpocetine inhibits VSMCs chemokinesis. A: Representative chemokinesis of VSMCs among the different groups. Wound closure was induced by normal (5 mM) or higher glucose (10, 25 mM) at 0, 24 and 48 h post-wounding in the absence or presence of vinpocetine (15, 30 and 50 µM). B: Statistical analysis of average gap areas relative to those measured at 0 h after wounding. Experiments were performed in triplicate. Bars indicate means ± SD. * means P<0.05 compared to control; # means P<0.05 compared to HG (25 mM) stimulation.

To examine the potential effect of vinpocetine on migration of VSMCs, a wound healing assay was performed. Quantification of the gap areas relative to those measured at 0 h after wounding was used to estimate chemokinesis of cells. As illustrated in Figure 3, migration of VSMCs was enhanced by incubation with 10 mM glucose for 48 h, as well as by 25 mM glucose stimulation for 24 h or 48 h. Nevertheless, vinpocetine treatment at higher concentration (30 or 50 µM) increased gap area at either 24 h or 48 h when compared to the HG (25 mM) groups.

### Vinpocetine attenuates HG-induced ROS generation

**Figure 4:**
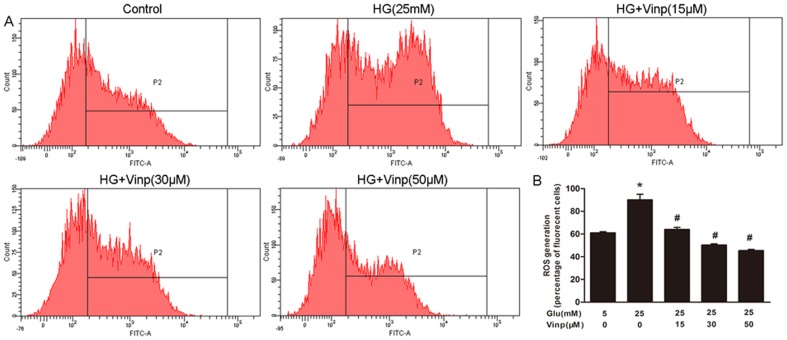
Vinpocetine attenuates ROS generation in VSMCs. A: Representative ROS generation in the different groups. The level of intracellular ROS production was detected by H2DCFDA fluorescence, using flow cytometry at excitation of 488 nm and emission of 528 nm. B: Analysis of H2DCFDA positive cells in percentage. Data are shown as the means ± SD of four experiments. * means P<0.05 compared to control; # means P<0.05 compared to HG (25 mM) stimulation.

An increase in ROS production was detected in HG-stimulated VSMCs, which was inhibited by vinpocetine treatment (Figure 4). Twenty five mM HG significantly increased ROS production from 61.0 ± 1.4% in the control group to 90.1 ± 6.9% (P<0.05). However, vinpocetine treatment markedly suppressed ROS generation at concentrations of 30 µM (50.4 ± 1.4%) and 50 µM (45.5 ± 1.4%) when stimulated by 25 mM glucose (P<0.05). Although with similar potential, no significant decrease was observed in the group that underwent vinpocetine treatment at a concentration of 15 µM.

### Vinpocetine induces apoptosis in the presence of HG

**Figure 5:**
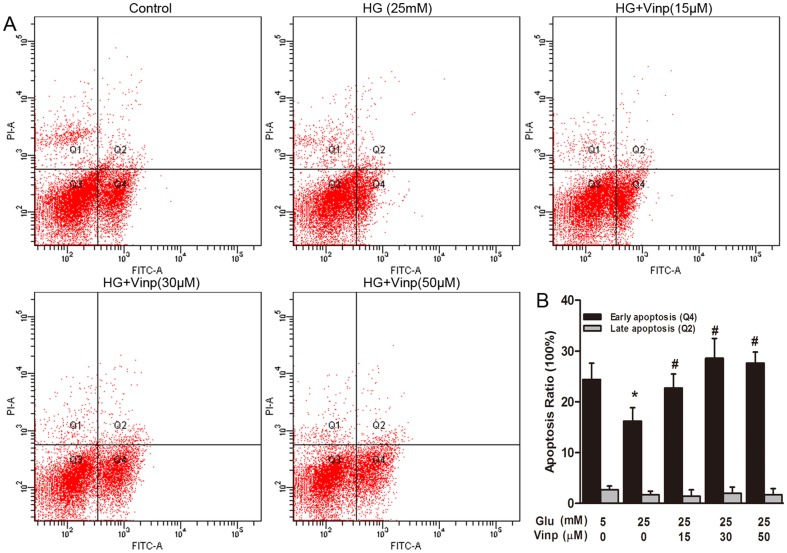
Vinpocetine induced early apoptosis in VSMCs. Apoptosis induced by FBS deprivation in different groups was analyzed using Annexin V-FITC/PI flow cytometry. A: Representative images of apoptosis analysis. In each plot, viable cells are in the Q3 quadrant, early apoptotic cells are in the Q4 quadrant and necrotic or late apoptotic cells are in the Q2 quadrant. B: Quantitative analysis of apoptotic cells in percentage. Data are shown as the means ± SD of three experiments. * means P<0.05 compared to control; # means P<0.05 compared to HG (25 mM) stimulation.

Serum deprivation induced apoptosis was detected in all groups (Figure 5). A concentration of 25 mM HG significantly attenuated early apoptosis from 24.4 ± 3.2% in the control group to 16.2 ± 2.6% (P<0.05). Vinpocetine induced early apoptosis at concentrations of 15 µM (22.7 ± 2.7%), 30 µM (28.6 ± 3.8%) and 50 µM (27.6 ± 2.2%) in the presence of HG stimulation (P<0.05). To assess the role of vinpocetine in VSMC apoptosis in vivo, cross-sections of the carotid arteries from different groups were examined using the TUNEL method. However, we did not find a significant increase in the percentage of TUNEL-positive cells in the neoinitima from vinpocetine-treated animals compared with saline-treated animals (Figure S1).

### Vinpocetine blocks PCNA, cyclin D1 and BCL-2 protein expression

**Figure 6:**
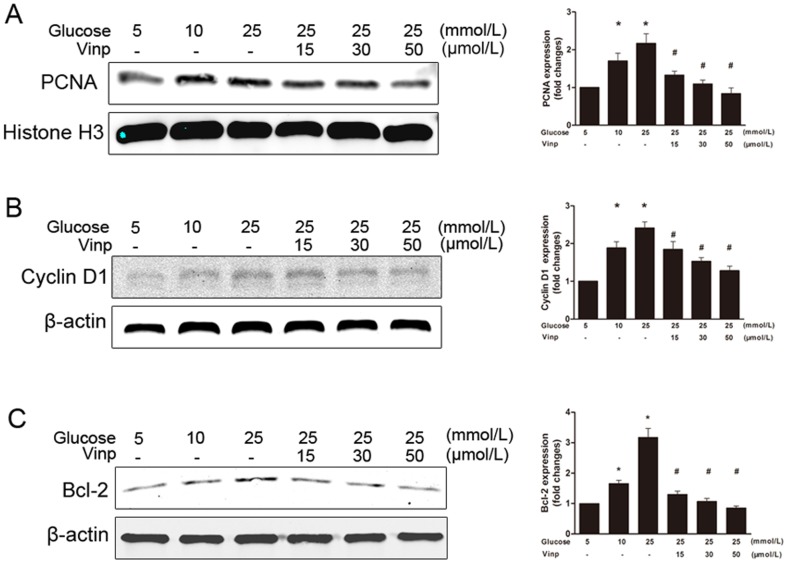
Vinpocetine blocks HG-stimulated PCNA, cyclin D1 and BCL-2 expression. The pictures represent immunoblots of proteins and the quantitative analysis of the immunoblot results. The density of each band was normalized to its own internal control (β-actin or histone H3 level). Data were expressed as fold changes compared with the control. A: Vinpocetine inhibits HG-stimulated PCNA expression. B: Vinpocetine suppresses HG-stimulated cyclin D1 expression. C: Vinpocetine attenuates HG-enhanced Bcl-2 expression. The values are mean ± SD. More than 3 independent experiments were replicated. * means P<0.05 compared to control; # means P<0.05 compared to HG (25 mM) stimulation.

**Figure 7:**
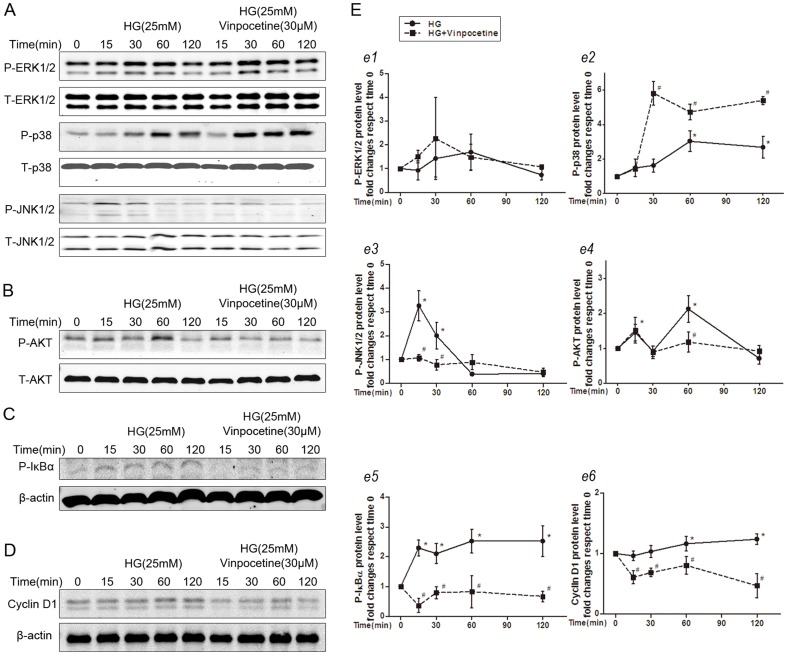
Vinpocetine regulates HG-induced cell signaling. A: Representative immunoblots of ERK1/2, p38, JNK phosphorylation and their own total protein levels. B: Vinpocetine prevents HG-stimulated PI3K/Akt activation. C: Vinpocetine inhibits HG-induced IκBα activation. D: Vinpocetine blocks cyclin D1 protein expression. E: Quantitative analysis of the immunoblot results. Each band density was normalized to its own internal control. Data were expressed as fold changes compared with the time 0 control. More than 3 independent experiments were replicated. * means P<0.05 compared to control; # means P<0.05 compared to HG (25 mM) stimulation.

Exposure to 25 mM glucose for 24 hours increased the expression of PCNA, cyclin D1 and Bcl-2 in cultured VSMCs. Vinpocetine treatment resulted in the inhibition of HG-induced expression of PCNA, cyclin D1 and Bcl-2 proteins in a dose-dependent manner (Figure 6A, B, C). A similar trend in cyclin D1 expression was observed in a time-dependant manner during the first 2 hours of vinpocetine treatment (Figure 7D).

### Vinpocetine regulates HG-mediated MAPK singling pathway

To clarify the effects of vinpocetine on the MAPK signaling pathway in VSMCs growth, individual MAPK phosphorylation was investigated. HG induced a rapid activation of ERK1/2, p38, and JNK1/2 without affecting their total levels (Figure 7A). As shown in Figure 7A, vinpocetine treatment significantly suppressed HG-induced JNK1/2 activation but not ERK1/2 activation. In contrast, p38 phosphorylation was markedly enhanced by vinpocetine treatment.

### Vinpocetine attenuates HG-mediated PI3K/Akt signaling

Akt activation was evaluated to investigate whether vinpocetine affected the PI3K/Akt pathway in VSMCs. As shown in Figure 7B, HG-induced phosphorylation of AKT was significantly enhanced at 15 min and 60 min, and this effect was completely suppressed by vinpocetine treatment.

### Vinpocetine blocks NF-κB-dependent protein expressions

We also investigated whether vinpocetine affected the NF-κB signaling pathway in the presence of HG stimulation. As shown in Figure 7C, HG significantly stimulated IκBα phosphorylation, which is required for NF-κB activation, and this activation was largely attenuated in vinpocetine-treated cells (Figure 7C).

## Discussion

Diabetes markedly increases the risk of coronary, cerebral, and peripheral atherosclerosis and the clinical consequences of myocardial infarction, stroke, limb ischemia, and death [14]. Revascularization is often necessary for severe atherosclerosis to avert the risk of end-organ damage [14]. Accumulation of abnormal VSMCs in the arterial wall plays a pivotal role in the formation of atherosclerotic lesions and in-stent restenosis in diabetics [15], [16].

Here, we demonstrated for the first time that vinpocetine inhibits high glucose-induced vascular smooth muscle cell migration, proliferation and apoptotic resistance in vitro and attenuates neointimal hyperplasia in balloon-injured diabetic rat carotid arteries in vivo. These beneficial effects of vinpocetine on VSMCs are associated with inhibition of ROS production and interference of the MAPK, PI3K/Akt, NF-κB signaling pathways as well as antiapoptotic protein expression. These findings suggest that vinpocetine may be a potential therapy for preventing injury-induced vascular remodeling in diabetes.

An animal model of diabetes and balloon injury was employed in the in vivo part of our study. Previous studies have indicated that STZ-induced diabetes characterized by hyperglycemia and hypoinsulinemia cannot thicken neointima in response to arterial injury [17], [18]. However, another study [19] has shown that prolonging the time interval between STZ injection and the injury procedure resulted in neointimal expansion, indicating that hyperglycemia can promote restenosis independent of hyperinsulinemia and insulin resistance. To avoid the effects of hypoinsulinemia and focus on the efficacy of vinpocetine on hyperglycemia facilitating vascular remodeling, we utilized low dose STZ injection (35 mg/kg) following 2-week HFD to model diabetic rats, which produced hyperglycemia in the presence of circulating insulin concentration comparable to normal rats [12]. Furthermore, to imitate the clinical procedure most closely, PTCA balloons replacing 2 F Fogarty embolectomy catheters were used in the in vivo part of our study.

Chronic hyperglycemia facilitates the development and progression of vascular pathology via multiple mechanisms. Consistent with previous studies [11], [19], our data demonstrated that the loss of the lumen and the I/M ratio was dramatically aggravated in diabetic animals. In addition, we found that vinpocetine treatment significantly alleviated neointimal hyperplasia at the injury site. More importantly, our data showed that vinpocetine attenuated the increase in PCNA-positive cells in the neointimal region and inhibited neointimal hyperplasia to a greater extent in diabetic rats than that in normal rats. The results indicated that vinpocetine slows down the development of restenosis after PCI, especially in patients with diabetes.

It is well documented that VSMC migration and proliferation play a pivotal role in the development of post-angioplasty restenosis [4]. In response to injury, VSMCs exhibit phenotypic plasticity, changing from a quiescent and contractile phenotype to a synthetic one [20]. Over the first 2 weeks after injury, the VSMCs multiply 3 to 5 times, accounting for 90% of the ultimate intimal proliferation [4]. Although without direct mitogenic effects on VSMCs [15], HG is believed to promote growth by enhancing the response to growth factor or cytokine stimulation [19]. Our early study[13] and other studies have shown that HG enhanced VSMCs migration and proliferation [13], [21]-[23]. In the present study, the EdU assay detected that VSMCs proliferation was stimulated by HG in the presence of 0.5% FBS, and vinpocetine significantly inhibited this proliferative effect in a dose-dependent manner. A previous study reported that vinpocetine inhibited PGDF-BB-induced VSMCs migration and proliferation [11]. Our data revealed that vinpocetine had the same potential in the presence of HG stimulation.

In addition to proliferation, apoptosis of VSMCs also participated in the pathogenesis and progression of macrovascular disease [24], [25]. HG could significantly attenuate apoptosis in response to serum withdrawal in cultured VSMCs [16], [26]. A recent study [10] revealed the potential of vinpocetine to induce apoptosis in breast cancer cells. We also tested the effects of vinpocetine on apoptosis in VSMCs. Consistent with previous studies, our data showed that HG protected VSMCs from apoptosis induced by serum deprivation. In addition, we are the first to report that vinpocetine aggravated apoptosis in the presence of HG. This implies that vinpocetine attenuated restenosis in diabetics by disturbing the balance between proliferation and apoptosis of VSMCs. Moreover, VSMCs prevented apoptosis through compensatory mechanisms when treated by anti-proliferative agents such as paclitaxel [27]. Our observation suggested that vinpocetine might be a more efficient agent for patients with diabetes. However, the slight between-group difference in TUNEL-positive cells suggested that environmental exposure throughout the experiments might influence the apoptotic state in vivo. Therefore, dynamic measurements of VSMC apoptosis in animal research should be applied to the future studies.

There is considerable evidence that an increased level of ROS is responsible for hyperglycemia-induced VSMC proliferation [13], [28]. Hyperglycemia-induced ROS production triggers several cellular mechanisms including polyol and hexosamine flux, protein kinase C (PKC) activation, and NF-kB-mediated vascular inflammation [29], which further stimulates VSMCs migration and proliferation [13]. Therefore, we investigated whether the antiproliferative effect of vinpocetine is mediated through the inhibition of ROS generation. Our data showed that vinpocetine significantly inhibited ROS overproduction in vitro. Consistent with our study, Cai et al. also found that vinpocetine could prevent PDGF-BB-induced ROS generation [11]. Among the various possible sources, growing evidence has suggested that NADPH oxidase may be the most important source of ROS production in vascular cells [30]. Further studies are needed to investigate the connection between vinpocetine and NADPH oxidase.

Regarding the intracellular mechanism, a growing body of evidence has suggested that ROS plays an important role in the activation of MAPK and PI3K/Akt cascades mediated by HG [13], [31]. We examined the MAPK signaling pathway to elucidate the effects of vinpocetine on intracellular signaling further. Consistent with previous reports [13], a marked activation of p38, ERK1/2, and JNK1/2 occurred in HG-stimulated VSMCs. Interestingly, only the phosphorylation of JNK1/2 was significantly inhibited by vinpocetine, indicating that activation of JNK1/2 plays an important role in the vinpocetine-mediated inhibitory effects on VSMCs proliferation and migration. In arterial VSMCs, p38 MAPK is considered a pro-apoptotic regulator [27]. Tsujimoto et al. [32] found that enhancement of apoptosis in VSMCs was associated with activation of p38. We found that phosphorylation of p38 and apoptosis were markedly enhanced by vinpocetine treatment, suggesting that activation of p38 plays an important role in the vinpocetine-enhanced VSMCs apoptosis.

PI3K/Akt signaling was reported to mediate cell survival, proliferation and migration of VSMCs [13], [33]. Our data revealed that vinpocetine blocked HG-stimulated Akt phosphorylation, indicating that the PI3K/Akt signaling pathway is also involved in vinpocetine-mediated inhibition of cell proliferation and chemokinesis. The downstream targets of the MAPKs and PI3K/Akt pathway include transcription factors, such as NF-κB, which is activated in vascular cells of diabetic rats and mice [34], and has been linked to proliferation and apoptosis in VSMCs [13], [35], [36]. In this study, we also provided evidence that vinpocetine inhibited HG-induced activation of NF-κB in VSCMs, causing IκBα phosphorylation, which is necessary for NF-κB activation, was significantly down-regulated by vinpocetine treatment. In fact, a previous study clearly demonstrated the inhibitory effect of vinpocetine on the NF-κB pathway [11]. Coupled with the present observations, we suggest that the NF-κB pathway is involved in vinpocetine-mediated VSMCs growth in the presence of HG. Of note, our data did not directly prove that vinpocetine affected the activity of NF-κB. Further work is required to test the efficacy of vinpocetine on the activity of NF-κB in the presence of HG.

To further the understanding of the downstream molecular mechanism, we investigated the PCNA protein expression. Consistent with our in vivo study, the HG-induced increase of PCNA expression was weakened by vinpocetine treatment. A similar trend was observed in cyclin D1 expression. Consistent with former studies [11], [13], [23], these results suggested that the antiproliferative effect of vinpocetine was in part due to the blockade of cell cycle progression through down-regulating cyclin D1. A previous study reported that HG suppressed VSMCs apoptosis by impacting Bcl-2 expression and caspase-3 activity [26]. Therefore, we were interested in investigating the involvement of apoptosis regulatory proteins on vinpocetine-enhanced apoptosis. Consistent with the increase in apoptosis, we found that the expression of antiapoptotic protein Bcl-2 was decreased. These data suggested that vinpocetine might attenuate HG-enhanced apoptotic resistance in VSMCs through down-regulating antiapoptotic protein expression.

## Conclusions

In conclusion, this study demonstrates that vinpocetine ameliorates hyperglycemia-facilitated neointimal formation in vivo and attenuates HG-stimulated VSMCs chemokinesis, proliferation and apoptotic resistance in vitro by blocking ROS production and affecting the MAPK, PI3K/Akt, and NF-κB signaling pathways. Therefore, vinpocetine may be an effective agent for delaying intimal hyperplasia and restenosis in diabetic vessels.

## Supporting Information

Figure S1 Vinpocetine does not affect apoptosis in neointima after balloon injury in vivo. Representative images (A) and statistical results (B) of apoptosis quantified as percentage of terminal deoxynucleotidyl transferase dUTP nick-end labeling (TUNEL)-positive cells within the neoinitma. Results were obtained in three independent experiments. * means P<0.05 compared to Sham.
(TIF)
