# Supplementary figures and images for: Vinpocetine Attenuates Neointimal Hyperplasia in Diabetic Rat Carotid Arteries after Balloon Injury
Source: PLoS One. 2014 May 12;9(5):e96894. doi: 10.1371/journal.pone.0096894 (PMC4018422; doi:10.1371/journal.pone.0096894)

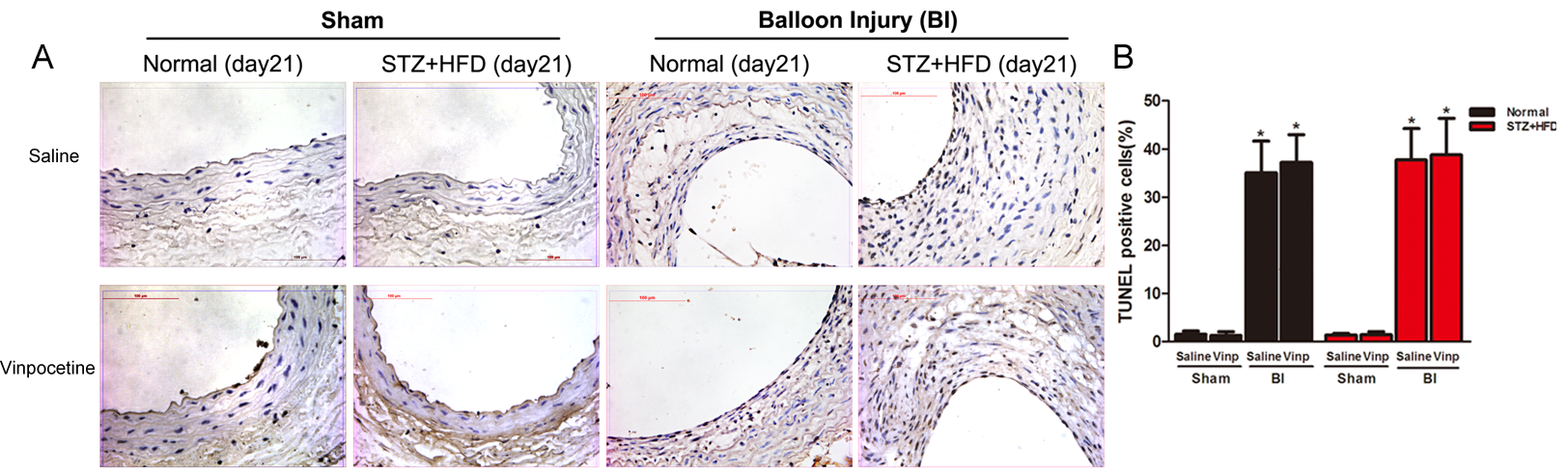

Supplement: Figure S1 — Vinpocetine does not affect apoptosis in neointima after balloon injury in vivo. Representative images (A) and statistical results (B) of apoptosis quantified as percentage of terminal deoxynucleotidyl transferase dUTP nick-end labeling (TUNEL)-positive cells within the neoinitma. Results were obtained in three independent experiments. * means P<0.05 compared to Sham. (TIF) [file pone.0096894.s001.tif]
